# Evaluating the Biochemical and Haematological Safety of the *Histoplasma capsulatum* var. *farciminosum* ‘8ZH’ Vaccine in Foals

**DOI:** 10.1002/vms3.70764

**Published:** 2026-01-07

**Authors:** Sabira E. Alpysbayeva, Akbope A. Abdykalyk, Kali Tileukhanov, Azamat R. Abdimukhtar, Alinur T. Toleukhan, Makhpal K. Sarmykova, Aktoty M. Anarbekova, Yeraly A. Shayakhmetov, Nazym S. Syrym, Sergazy Sh. Nurabaev, Bolat A. Yespembetov

**Affiliations:** ^1^ Research Institute for Biological Safety Problems National Holding “QazBioPharm Otar Kazakhstan; ^2^ Al‐Farabi Kazakh National University Almaty Kazakhstan

**Keywords:** biochemical markers, biochemical parameters, epizootic lymphangitis, equine vaccine, foals, histoplasma farciminosum, histoplasma capsulatum var. farciminosum, vaccine safety

## Abstract

**Background:**

Epizootic lymphangitis (EEL), caused by *Histoplasma capsulatum* var. *farciminosum* (HCF), is a neglected equine fungal disease lacking effective vaccines. The newly developed inactivated ‘8ZH’ vaccine requires safety validation in the target species.

**Objectives:**

To evaluate the biochemical, haematological and clinical safety of the inactivated HCF ‘8ZH’ vaccine in foals.

**Methods:**

A controlled, single‐blinded study was conducted on 30 clinically healthy foals (4–6 months), randomized into vaccinated (n = 15) and control (n = 15) groups. Vaccinated animals received a 5 mL intramuscular dose (10 mg antigen, MONTANIDE GEL 01 PR adjuvant) on Day 0 and a booster on Day 21. Clinical observations (temperature, appetite, behaviour, injection site) were recorded daily. Blood was collected at baseline and on Days 7, 14, 21, 35 and 42 for biochemical, haematological and acute‐phase protein analysis. Data were analysed using repeated measures ANOVA.

**Results:**

No severe or adverse reactions were observed. Mild, transient injection‐site swelling (< 4 cm) occurred in three vaccinated foals and resolved spontaneously. All animals maintained normal temperature and appetite. AST showed a transient increase on Day 14 (*p* = 0.04); WBC also rose (*p* = 0.03), indicating a typical immune response. Other parameters, including ALT, GGT, creatinine, total bilirubin and acute‐phase proteins (SAA, fibrinogen, haptoglobin), remained within physiological limits. No statistically significant long‐term deviations or toxic effects were noted.

**Conclusions:**

The inactivated HCF ‘8ZH’ vaccine demonstrated a favourable safety profile in foals. These results support its continued development for use in EEL prevention programs.

## Graphical abstract

This study assessed the biochemical and haematological safety of the inactivated *Histoplasma capsulatum* var. *farciminosum* ‘8ZH’ vaccine in foals. No adverse clinical signs or significant changes in liver, kidney or acute‐phase protein markers were observed. The results support the vaccine's safety and suitability for further immunogenicity evaluation in equine epizootic lymphangitis control.

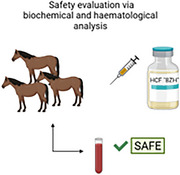

## 1 Introduction

Epizootic lymphangitis (EEL) is a chronic and highly contagious fungal disease in equines, caused by *Histoplasma capsulatum* var. *farciminosum* (HCF), a thermally dimorphic variety of *Histoplasma capsulatum* (Ameni 2007). The disease predominantly impacts horses, although donkeys and mules exhibit enhanced resistance (Selim et al. 1985; Al‐Ani and Al‐Delaimi 1986). EEL is characterized by granulomatous lesions in the lymphatic vessels, skin and lungs, posing significant risks to equine health, productivity and welfare, especially in areas with limited veterinary access (Scantlebury & Reed, 2009). The disease persists as endemic in various African countries (Ethiopia, Nigeria, Sudan, Senegal, South Africa) (Abdela et al. 2021; Ameni 2006; Addo 1980; Abdullahi et al. 2019; Awad 1960), the Middle East (Egypt, Iraq) (Hamid and Yousif 2001), and certain regions of Asia, including Kazakhstan (Kaisenov et al., 2024).

Vaccination is widely considered the most effective strategy for controlling EEL; however, no commercially available vaccines currently exist. The first vaccine, developed using the T21 strain, was tested in China and demonstrated 75.5% protective efficacy (Zhang et al. 1986). Although Al‐Ani's formalin‐inactivated vaccine has been proposed, a comprehensive production methodology is not yet available (Al‐Ani 1999). In response to recent outbreaks in Northern Kazakhstan, a new inactivated vaccine, HCF ‘8ZH’, was developed as part of ongoing research efforts (Kazinform, 2024).

While previous research has primarily focused on the immune response and protective effects of HCF vaccines, little is known about their influence on biochemical and haematological parameters in foals. Understanding these effects is important, as vaccine‐induced metabolic or haematological changes may have implications for foal health and development. This study investigates such responses following administration of the ‘8ZH’ vaccine, offering new insights into its safety profile. By addressing this gap, our findings aim to inform decisions regarding the vaccine's potential role in equine health management and EEL control strategies.

## 2 Methods

### 2.1 Study Design

**FIGURE 1:**
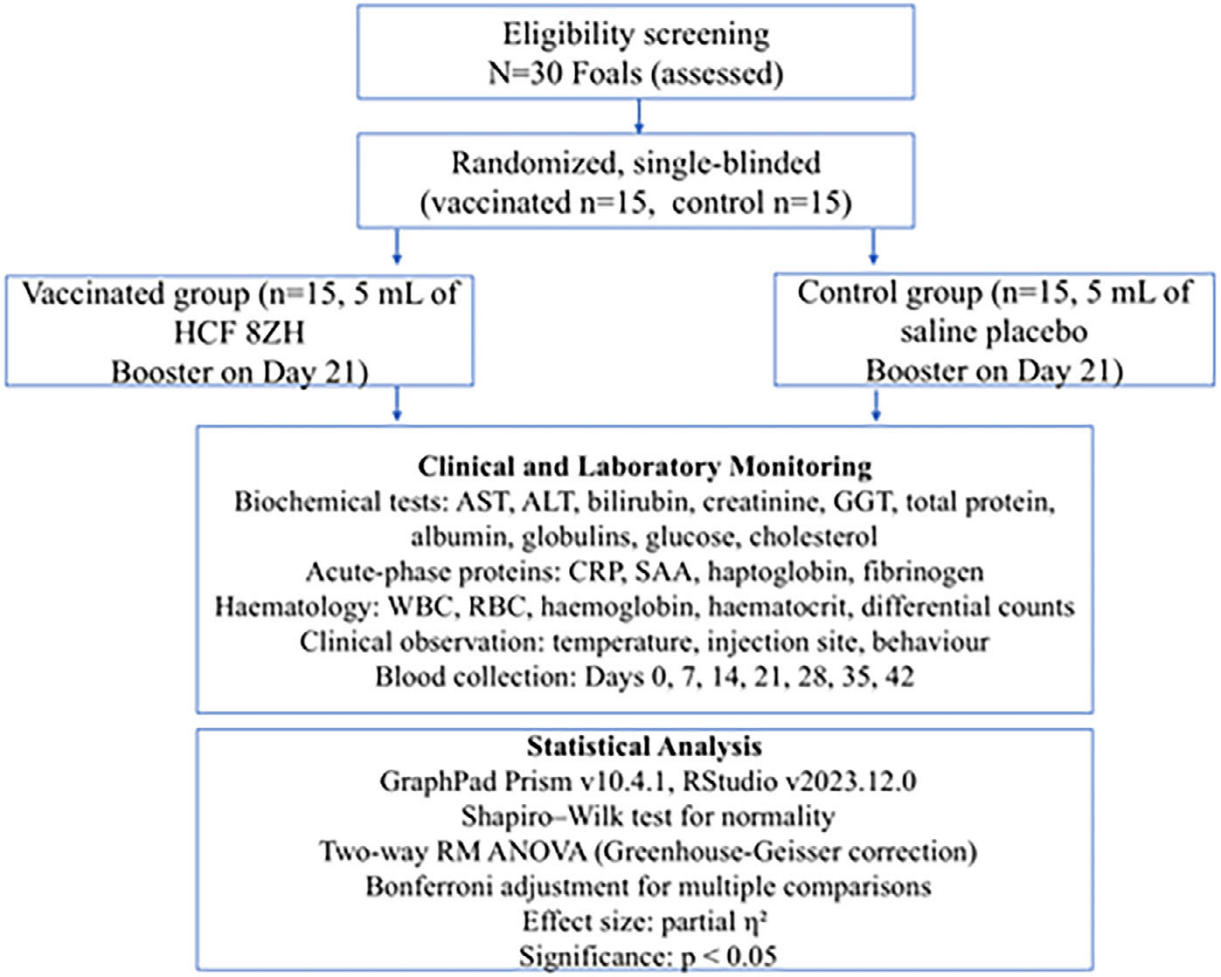
Flowchart of the study design.

This study was conducted as a randomized, controlled, single‐blinded trial to evaluate the biochemical and haematological responses of foals to the HCF ‘8ZH’ vaccine. Thirty clinically healthy foals aged 6–8 months were randomly assigned to either a vaccinated group (*n = *15) or a control group (*n = *15). The vaccinated group received 5 mL of the inactivated HCF vaccine intramuscularly in the neck region, with a booster dose on Day 21. The control group received 5 mL of sterile saline under identical conditions. Laboratory personnel performing biochemical and haematological analyses were blinded to group allocation to reduce bias (Figure 1).

### 2.2 Subjects, Screening and Housing

Foals were acquired from a private breeder and had no documented exposure to HCF. Before inclusion, all animals received a 14‐day quarantine during which a full physical examination was conducted to exclude previous exposure to HCF.

An agar gel immunodiffusion assay (AGID), routinely utilized in our laboratory and validated using reference sera, was employed to verify the lack of anti‐HCF antibodies.

The inclusion criteria were: (1) age between 6 and 8 months, (2) clinical health based on veterinary examination, (3) no prior antifungal therapy or indications of mycosis and (4) negative AGID results for HCF.

The sex distribution was nearly same across groups, with 8 males and 7 females in the vaccinated group, and 7 males and 8 females in the control group. All foals were maintained under uniform conditions with unrestricted access to water and a standardized feeding protocol. The research adhered to ARRIVE guidelines and received approval from the Institutional Animal Care and Use Committee (Ethics Protocol No. 1, approved 23 July 2023).

### 2.3 Vaccine Formulation and Administration 8810315716

The inactivated vaccine was based on the epizootic strain HCF ‘8ZH’, isolated from an equine outbreak in Pavlodar, Northern Kazakhstan (Tileukhanov et al., 2023). The strain was cultured in Sabouraud Dextrose Broth (Condalab) at 28°C for 7 days. Inactivation was performed in two stages:

- Chemical Inactivation: The fungal culture was treated with β‐propiolactone (BPL, Thermo Fisher Scientific) to a final concentration of 0.05% and incubated at 4°C–8°C for 6 h.
- Ultrasonic Disintegration: The treated biomass (500 mL) was subjected to 30 min of ultrasonic homogenization (Hielscher UP400St, 22 kHz, 1000 W/cm^2^) on ice. The suspension was then held at +2°C to +8°C overnight, centrifuged at 10,000 rpm for 20 min and filtered through a sterile gauze mat.

### 2.4 Antigen Quantification and Purity Assessment

Prior to formulation, the concentration of total soluble protein in the inactivated HCF (‘8ZH’) suspension was determined using the Bradford protein assay (Bio‐Rad, USA), with bovine serum albumin (BSA) as the standard. Based on these measurements, the antigen dose was adjusted to 2 mg/mL, corresponding to 10 mg of protein per 5 mL vaccine dose.

#### 2.4.1 SDS‐PAGE and Western Blotting

The purity of the antigen was assessed using SDS‐PAGE on 12% polyacrylamide gels under reducing conditions. Gels were stained with Coomassie Brilliant Blue to visualize protein bands. To confirm antigen identity, Western blotting was performed with hyperimmune equine serum as the primary antibody.

Proteins were transferred to PVDF membranes and blocked with 5% non‐fat dry milk in TBS‐T (Tris‐buffered saline with 0.1% Tween‐20) for 1 h at room temperature. Membranes were incubated overnight at 4°C with the primary antibody. After washing, they were treated with HRP‐conjugated goat anti‐horse IgG (Jackson ImmunoResearch), validated for Western blot applications.

Detection was carried out using enhanced chemiluminescence (ECL; Bio‐Rad, USA), and signals were visualized with a ChemiDoc XRS+ imaging system.

#### 2.4.2 Batch‐to‐Batch Consistency

Three separate production lots of the inactivated antigen were analysed by SDS‐PAGE to assess batch‐to‐batch reproducibility.

#### 2.4.3 Sterility Testing

Sterility of the final vaccine formulation was confirmed by culturing aliquots on Sabouraud Dextrose Agar and Brain Heart Infusion Broth (Sigma‐Aldrich) at 28°C and 37°C for 14 days. No microbial growth was observed.

#### 2.4.4 Final Vaccine Composition and Administration

The final vaccine formulation contained 10 mg of inactivated *H. capsulatum* antigen per 5 mL dose (2 mg/mL), MONTANIDE GEL 01 PR (10%) (Seppic, France) as an adjuvant, and thiomersal (0.01%) as a preservative. The pH was adjusted to 7.2–7.6 using NaOH. Foals in the vaccinated group received a 5 mL intramuscular injection, while control animals received 5 mL of sterile saline. A booster was administered to all animals on Day 21.

### 2.5 Sample Size Justification

An a prior power analysis was conducted using G*Power 3.1. To detect a 20%–30% difference in AST values (SD = 10–15 U/L), the required sample size was estimated at 176–394 animals per group. AST was chosen as a surrogate marker for power estimation given its established sensitivity to hepatic perturbations in toxicological and vaccine studies Due to ethical and logistical limitations typical of early‐phase vaccine trials in large animals, a pilot design was implemented using 15 animals per group. This approach is supported by published preliminary studies in equine vaccine safety (Allkofer et al. 2021; El‐Hage et al. 2022; Santos et al. 2021), which often use small cohorts for initial safety screening before scaling up to efficacy trials (Wagner et al. 2015).

### 2.6 Biochemical and Haematological Assessments

Blood samples were collected from each animal at seven time points: before immunisation (Day 0), and on Days 7, 14, 21, 28, 35 and 42 post‐vaccination. Approximately 8 mL of whole blood was drawn via jugular venipuncture into plain and EDTA‐containing tubes.

Biochemical analyses were performed on serum using the IDEXX Catalyst One analyzer. The panel included liver enzymes (AST, ALT), total protein, albumin, calculated globulins, total bilirubin, creatinine, blood urea nitrogen, glucose, cholesterol and acute‐phase proteins (fibrinogen, C‐reactive protein [CRP], serum amyloid A [SAA] and haptoglobin).

Haematological parameters were assessed using the IDEXX ProCyte Dx analyzer and included total leukocyte and erythrocyte counts, haemoglobin concentration, haematocrit and leukocyte differentials (neutrophils, lymphocytes, monocytes and eosinophils).

Alongside laboratory evaluations, clinical observations were conducted at each sampling point. Monitoring included rectal temperature, local inspection of the injection site for erythema, swelling, or induration, as well as general behaviour, appetite and findings on physical examination. All evaluations were carried out by the same veterinarian to ensure consistency. No sedation or anaesthesia was required for sample collection, and animals tolerated the procedures well throughout the study period.

### 2.7 Statistical Analysis

Data were analysed using GraphPad Prism (v10.4.1) and RStudio (v2023.12.0). Normality was assessed via the Shapiro‐Wilk test. Time‐dependent effects were evaluated using two‐way repeated measures ANOVA. The Greenhouse‐Geisser correction was applied when the assumption of sphericity was violated.

The use of repeated measures ANOVA was selected over mixed models due to the balanced study design, consistent sampling intervals and fixed group structure. While more complex models may offer marginally greater flexibility, ANOVA remains a validated and interpretable method for controlled experimental designs of this scale. Bonferroni correction was applied for multiple comparisons. Effect sizes were reported as partial eta squared (η^2^), with statistical significance set at *p *< 0.05 and 95% confidence intervals included for key estimates.

## 3 Results

### 3.1 Antigen Purity and Identity Analysis

The inactivated HCF (‘8ZH’) antigen showed acceptable purity and immunoreactivity, as confirmed by SDS‐PAGE and Western blot analysis (Figure S1). A notable immunoreactive band of about 15 kDa was consistently seen in all three production batches utilising hyperimmune equine serum and HRP‐conjugated goat anti‐horse IgG. This band presumably signifies a conserved low‐molecular‐weight epitope that persisted after β‐propiolactone deactivation and ultrasonic disruption. Although additional faint bands between 25–50 kDa were observed in certain technical replicates, their inconsistent presence is likely attributable to fluctuations in protein loading or blotting efficiency rather than genuine antigenic discrepancies. No control blot utilising naïve serum was conducted, hence non‐specific binding cannot be entirely discounted. The consistent existence of the predominant 15 kDa signal across batches supports the immunological significance and reliability of the antigen preparation. Sterility testing verified the lack of microbiological contamination during 14 days of culture, signifying that the finished product was safe for formulation and utilisation.

### 3.2 Biochemical and Clinical Assessments

Throughout the observation period, all foals remained clinically healthy. No systemic adverse reactions were observed following administration of the inactivated vaccine. Physical examinations were conducted daily to assess body temperature, general behaviour, appetite and local injection site reactions. Body temperatures remained within physiological limits (range: 37.5°C–38.9°C), and all animals maintained normal feeding behaviour and activity levels.

Due to the formulation volume (5 mL per dose), injection sites were carefully examined for local inflammation. No signs of heat, pain or lameness were detected. In three foals, mild, diffuse swelling (< 4 cm in diameter) was observed within 24–48 h post‐injection. These swellings were non‐painful and resolved spontaneously within 48 h without intervention. Such findings are consistent with normal local responses to larger‐volume intramuscular vaccinations and are not indicative of adverse inflammatory pathology.

Biochemical parameters in the vaccinated group demonstrated overall stability and remained within established physiological reference ranges, supporting the vaccine's safety profile (Table 1).

**TABLE 1:** Full biochemical data over time (all values presented as mean ± SD).

			Experimental group (*n* = 10)					
Parameter	Reference range	Control group (*n* = 10)	Pre‐vaccination	Day 7	Day 14	Day 21	Day 35	Day 42
Total protein (g/L)	55–75	69.2 ± 0.01	60.1 ± 0.01	63.0 ± 0.02	68.0 ± 0.03	65.0 ± 0.03	63.0 ± 0.27	62.0 ± 0.27
Albumin (g/L)	27–39	32.0 ± 0.4	32.5 ± 0.5	31.8 ± 0.6	31.8 ± 0.6	31.8 ± 0.6	31.8 ± 0.6	31.8 ± 0.6
Globulins (g/L)	18–34	28.2 ± 0.5	28.2 ± 0.5	28.2 ± 0.5	28.2 ± 0.5	28.2 ± 0.5	28.2 ± 0.5	28.2 ± 0.5
Fibrinogen (g/L)	1.0–3.5	1.9 ± 0.1	1.9 ± 0.1	1.9 ± 0.1	1.9 ± 0.1	1.9 ± 0.1	1.9 ± 0.1	1.9 ± 0.1
SAA (mg/L)	< 20	9.0 ± 1.2	9.0 ± 1.2	9.0 ± 1.2	9.0 ± 1.2	9.0 ± 1.2	9.0 ± 1.2	9.0 ± 1.2
Haptoglobin (mg/dL)	20–100	34.0 ± 3.0	34.0 ± 3.0	34.0 ± 3.0	34.0 ± 3.0	34.0 ± 3.0	34.0 ± 3.0	34.0 ± 3.0
Total bilirubin (mg/dL)	5–40	14.5 ± 0.7	27.3 ± 0.2	25.3 ± 0.6	23.8 ± 0.47	24.1 ± 0.51	25.1 ± 0.57	26.1 ± 0.57
AST (U/L)	116–287	201.0 ± 7.8	201.0 ± 7.8	205.3 ± 6.4	208.7 ± 7.21	207.5 ± 6.82	198.4 ± 5.29	210.4 ± 5.30
ALT (U/L)	< 30	1.5 ± 1.7	4.5 ± 0.54	5.2 ± 0.45	5.13 ± 0.38	4.9 ± 0.25	4.2 ± 0.29	4.7 ± 0.23
GGT (U/L)	10–60	24.0 ± 2.5	24.0 ± 2.5	24.0 ± 2.5	24.0 ± 2.5	24.0 ± 2.5	24.0 ± 2.5	24.0 ± 2.5
CRP mg/L	< 15	0.8 ± 0.2	0.5 ± 0.2	8.2 ± 1.5	17.4 ± 2.5	9.1 ± 1.3	4.5 ± 1.1	2.0 ± 0.8
Urea (mmol/L)	7–20	2.7 ± 0.5	3.9 ± 0.2	3.1 ± 0.19	2.9 ± 0.21	3.5 ± 0.3	4.1 ± 0.41	3.9 ± 0.4
Glucose (mmol/L)	3.9–7.5	3.9 ± 0.64	4.0 ± 0.7	4.1 ± 0.5	3.9 ± 0.62	3.1 ± 0.51	4.2 ± 0.72	4.0 ± 0.62
Creatinine (µmol/L)	77–175	130.0 ± 1.67	135.0 ± 1.67	135.0 ± 1.67	137.2 ± 1.71	139.1 ± 1.82	139.8 ± 1.78	138 ± 1.73
Cholesterol (mmol/L)	1.6–4.2	2.2 ± 1.6	2.3 ± 1.3	2.5 ± 1.8	2.58 ± 1.75	3.6 ± 2.12	2.41 ± 1.48	2.3 ± 1.58

*Note*: The reference intervals for SAA (< 20 mg/L), CRP (< 1.5–2.0 mg/L), fibrinogen (1.0–3.5 g/L), and haptoglobin (20–100 mg/dL) were based on studies in healthy foals and adult horses, as well as institutional guidelines (Jacobsen et al. 2004; Piccione et al. 2020; Crisman et al. 2008).

Following vaccination, liver enzyme activities, including AST, ALT and GGT, remained largely unaltered. AST levels exhibited a mild, transient elevation, peaking at 210.4 ± 5.3 U/L on Day 42, compared to baseline values of 201.0 ± 7.8 U/L. Despite this increase, values remained well within the normal range (116–287 U/L), and levels returned toward baseline, suggesting the absence of significant hepatocellular damage. ALT levels showed a modest post‐vaccination rise from 1.5 ± 1.7 U/L to 5.2 ± 0.45 U/L by Day 14, with no values approaching the threshold for clinical concern (< 30 U/L). GGT activity remained unchanged across all time points (24.0 ± 2.5 U/L), indicating preserved biliary function. A slight increase in total bilirubin concentration was noted, rising from 14.5 ± 0.7 mg/dL at baseline to 26.1 ± 0.57 mg/dL by Day 42, without exceeding the upper reference limit.

Renal parameters were similarly stable. Serum creatinine ranged from 130.0 ± 1.67 to 139.8 ± 1.78 µmol/L, and urea values fluctuated mildly between 2.7 ± 0.5 and 4.1 ± 0.41 mmol/L, with no indications of nephrotoxicity. Glucose concentrations remained within expected physiological limits (3.9–4.2 mmol/L), and cholesterol levels displayed only minor variation, with a peak of 3.6 ± 2.12 mmol/L on Day 35.

Regarding protein fractions, total protein levels declined slightly from 69.2 ± 0.01 g/L pre‐vaccination to 62.0 ± 0.27 g/L on Day 42, yet all values remained within the reference range (55–75 g/L). Albumin concentrations were stable across all time points (31.5–32.5 g/L), while globulin levels exhibited a modest increase, peaking at 30.2 ± 0.7 g/L on Day 14, potentially reflecting early‐phase immune activation.

Acute‐phase protein responses were evident but modest. Fibrinogen rose from 1.9 ± 0.1 to 2.5 ± 0.18 g/L by Day 14 before returning toward baseline, and SAA levels increased transiently, reaching 19.0 ± 2.4 mg/L before declining. Haptoglobin followed a similar trend, rising to a maximum of 56.0 ± 5.1 mg/dL during the second week post‐vaccination.

CRP also increased transiently following vaccination, rising from a baseline of 0.5 ± 0.2 mg/L to a peak of 17.4 ± 2.5 mg/L on Day 14, before returning to near‐baseline levels (0.8 ± 0.3 mg/L) by Day 42. Although this exceeded the typical reference range for healthy foals (< 1.5 mg/L), the self‐limiting elevation is consistent with normal acute‐phase responses to immunization, as reported in equine vaccine studies.

Across all parameters, differences between the vaccinated and control groups were not statistically significant (*p* > 0.05), indicating no vaccine‐associated biochemical toxicity.

### 3.3 Haematological Assessments

In the vaccinated group, haematological evaluations revealed a temporary increase in total white blood cell count, rising from 9.5 ± 0.6 × 10^9^/L at baseline to 11.8 ± 0.7 × 10^9^/L on Day 14 (*p* = 0.03), consistent with a short‐lived immunological response. By Day 28, WBC values had returned to near‐baseline levels (Table 2).

**TABLE 2:** Full haematological data over time (all values presented as mean ± SD).

			Experimental group (*n* = 10)					
Parameter	Reference range	Control group (*n* = 10)	Pre‐vaccination	Day 7	Day 14	Day 21	Day 35	Day 42
WBC (× 10^9^/L)	5.5–12.5	7.8 ± 0.5	7.9 ± 0.5	8.0 ± 0.6	8.2 ± 0.6	8.1 ± 0.5	7.9 ± 0.5	7.9 ± 0.5
RBC (× 10^1^ ^2^/L)	6.5–12.5	9.1 ± 0.4	9.2 ± 0.4	9.2 ± 0.3	9.3 ± 0.3	9.2 ± 0.3	9.1 ± 0.3	9.0 ± 0.3
Haemoglobin (g/L)	110–180	135 ± 5.2	136 ± 5.0	136 ± 4.8	138 ± 4.9	137 ± 4.7	136 ± 4.6	136 ± 4.6
Haematocrit (%)	32–52	41.5 ± 2.1	41.6 ± 2.0	41.8 ± 2.2	42.1 ± 2.3	41.9 ± 2.1	41.6 ± 2.0	41.6 ± 2.0
Neutrophils (%)	30–70	52.4 ± 4.1	52.5 ± 4.0	53.0 ± 3.8	53.7 ± 3.9	53.2 ± 3.7	52.8 ± 3.6	52.8 ± 3.6
Lymphocytes (%)	25–60	40.2 ± 3.8	40.3 ± 3.7	39.8 ± 3.6	39.5 ± 3.7	39.6 ± 3.5	40.0 ± 3.4	40.0 ± 3.4
Monocytes (%)	0–10	4.1 ± 1.2	4.1 ± 1.2	4.2 ± 1.3	4.3 ± 1.3	4.2 ± 1.2	4.1 ± 1.1	4.1 ± 1.1
Eosinophils (%)	0–8	3.3 ± 1.0	3.3 ± 1.0	3.4 ± 1.1	3.5 ± 1.1	3.4 ± 1.0	3.3 ± 0.9	3.3 ± 0.9

Red blood cell parameters, including haemoglobin concentration, haematocrit and erythrocyte count, remained within normal ranges throughout the study. No anaemia or abnormalities in erythrocyte indices were noted (*p* > 0.05).

Differential leukocyte counts further supported the interpretation of a self‐limiting vaccine response. Neutrophil percentages exhibited a non‐significant upward trend on Day 14 (*p* = 0.07), while lymphocyte and monocyte counts remained stable, indicating the absence of persistent systemic inflammation.

All haematological, biochemical and clinical findings are summarized in Tables 1 and 2. Comprehensive statistical outputs, including *p* values, effect sizes (η^2^) and confidence intervals, are provided in Tables S1 and S2.

## 4 Discussion

This study represents the first investigation into the biochemical and haematological safety of the HCF (‘8ZH’) inactivated vaccine in foals. To date, no published reports have assessed laboratory or clinical responses to vaccination against EEL, making these findings a valuable initial benchmark for vaccine safety evaluation in equine populations.

Overall, the vaccine demonstrated an excellent safety profile. Clinical monitoring showed no systemic adverse effects, with only mild and transient swelling observed in a few animals at the injection site. This aligns with expected local responses to intramuscular vaccination with a 5 mL volume and does not indicate abnormal reactogenicity.

Biochemical parameters remained within physiological limits throughout the study. Notably, alanine aminotransferase (ALT), bilirubin, creatinine, glucose and cholesterol did not show significant fluctuations, suggesting the absence of hepatic or renal toxicity. A transient rise in aspartate aminotransferase (AST) on Day 14 (*p *= 0.04) returned to baseline levels by Day 42, indicating a self‐limiting, localized muscular response to the vaccine. In our data, AST increased from 201.0 ± 7.8 U/L to 210.4 ± 5.3 U/L, remaining within the reference range (116–287 U/L) (Table 1). This modest and reversible shift is consistent with mild muscular irritation rather than hepatic damage. These results are in line with other equine vaccine studies, such as Allkofer et al. (2021), which documented transient, non‐pathological enzyme fluctuations following immunisation.

White blood cell (WBC) counts increased significantly on Day 14 (*p* = 0.03), reflecting an expected vaccine‐induced activation of the innate immune response. Similar transient leucocytosis has been reported in foals and adult horses after influenza and herpesvirus vaccinations (El‐Hage et al. 2022; Wagner et al. 2015). Stable erythrocyte indices and absence of anaemia further support the vaccine's haematological safety.

Importantly, this study also evaluated acute‐phase protein responses, which have not previously been reported in EEL vaccine trials. We observed a mild increase in fibrinogen and SAA, peaking at Day 14, and a parallel rise in haptoglobin during the second post‐vaccination week. Specifically, fibrinogen rose from 1.9 ± 0.1 to 2.5 ± 0.18 g/L, and SAA reached 19.0 ± 2.4 mg/L on Day 14, both remaining within or near reference limits. These markers normalized thereafter, supporting a regulated, non‐pathogenic acute‐phase response (Table 1).

In addition to these markers, CRP levels exhibited a transient elevation, peaking at 17.4 ± 2.5 mg/L on Day 14, exceeding the typical reference range for healthy foals (< 15 mg/L). However, CRP decreased to 9.1 ± 1.3 mg/L by Day 21 and returned near baseline by Day 42 (2.0 ± 0.8 mg/L). This dynamic is consistent with known physiological responses to vaccination and does not suggest pathological inflammation. Previous studies reported similar CRP elevations (15–25 mg/L) following equine influenza and herpesvirus immunization, without accompanying clinical abnormalities (Piccione et al. 2020; Biondi et al. 2022). Jacobsen et al. (2005) also demonstrated that CRP can rise significantly in response to immune stimuli in horses, even in the absence of disease. Crisman et al. (2008) reviewed the acute‐phase response in equine species and emphasized the diagnostic value of SAA, haptoglobin and CRP in detecting early, subclinical inflammation. Their findings support the interpretation that mild, transient elevations in these markers can occur following immune stimulation, including vaccination, without indicating pathological processes.

Furthermore, the absence of significant group differences (*p* > 0.05) across all measured parameters reinforces the conclusion that the ‘8ZH’ vaccine did not induce systemic biochemical or haematological toxicity.

Inclusion of antigen purity testing by SDS‐PAGE and Western blotting further addresses concerns regarding vaccine quality and batch consistency. A clear dominant band around ∼15 kDa was consistently detected, indicating stable antigen composition. Combined with sterility and protein quantification assays, these data strengthen the manufacturing reliability and reproducibility of the formulation.

These safety data provide a strong foundation for subsequent immunogenicity and efficacy studies, which will include evaluation of specific antibody titres and cellular immune responses to the HCF ‘8ZH’ vaccine.

### 4.1 Limitations and Future Directions

As a pilot safety trial, the study was limited by its relatively small sample size (*n* = 30). While this is consistent with early‐phase veterinary vaccine studies (Santos et al. 2021), rare adverse events may go undetected. Follow‐up trials with larger populations and longer observation periods are recommended to confirm long‐term safety and immunogenicity.

Additionally, while sex and age were balanced in the study design, a more detailed analysis of sex‐specific responses may be warranted, given known differences in immune regulation. The exclusion of animals with prior HCF exposure strengthens the study's internal validity, but generalization to endemic populations should be approached with caution.

Finally, future studies may benefit from longitudinal mixed‐effects models to account for individual variation and maximize statistical power.

## 5 Conclusion

The HCF ‘8ZH’ vaccine demonstrated a favourable biochemical and haematological safety profile in foals. Transient elevations in AST, CRP, fibrinogen and white blood cell counts were consistent with physiological vaccine responses and did not indicate systemic or organ‐specific toxicity. No severe local reactions or behavioural changes were observed. These findings provide foundational data for the development of an EEL vaccine and support its potential role in equine disease control strategies. Ongoing and future studies will assess its immunogenicity and duration of protection to determine suitability for broader application.

## Author Contributions

**Sabira E. Alpysbayeva**: supervision, project oversight, writing – review and editing. **Akbope A. Abdykalyk**: writing – original draft, statistical analysis. **Kali Tileukhanov**: investigation, literature review. **Azamat R. Abdimukhtar**: methodology, sample collection. **Alinur T. Toleukhan**: resources, ethics compliance. **Makhpal K. Sarmykova**: project administration, funding acquisition. **Aktoty M. Anarbekova**: laboratory work, data processing. **Yeraly A. Shayakhmetov**: laboratory work, experimental support. **Nazym S. Syrym**: institutional support, review and editing. **Sergazy Sh. Nurabaev**: institutional support, ethics compliance. Eldos Serikbay: sample preparation and data collection. Aslan Kerimbayev: study design and supervision. **Bolat A. Yespembetov**: corresponding author, final approval.

## Funding

This research was funded by the Research Institute for Biological safety problems, under the National holding “QazBioPharm”, Kazakhstan.

## Ethics Statement

This study was conducted following the ARRIVE guidelines and was approved by the Bioethics Commission of the Research Institute for Biological Safety Problems, National Holding “QazBioPharm,” Kazakhstan on 13 July 2023. The *H. farciminosum* ‘8ZH’ strain was isolated from an outbreak site in Northern Kazakhstan (Pavlodar) as part of a disease surveillance program conducted by licensed veterinarians. Field samples were collected from clinically affected equines following national veterinary regulations and institutional ethical guidelines.

## Consent

No client‐owned animals were used in this study. All animals were part of a controlled research population maintained under institutional veterinary supervision.

## Conflicts of Interest

The authors declare no conflicts of interest.

## Supporting information



Supplementary Figure 1. Western blot analysis of inactivated *Histoplasma capsulatum* var. *farciminosum* (‘8ZH’) antigen, Lane 1: Protein molecular weight marker (kDA); Lanes 2,3: Batch 1 (technical replicates); Lanes 4,5: Batch 2 (technical replicates); Lanes 6,7: Batch 3 (technical replicates)
Supplementary Table 1. Statistical analysis of biochemical parameters
Supplementary Table 2. Statistical analysis of hematological parameters
(p‐values, 95% CI, and effect sizes (η^2^) for all hematological parameters.)

## Data Availability

Bolat A. Yespembetov had full access to all the data in the study and takes responsibility for the integrity of the data and the accuracy of data analysis.
