# Supplementary material for: Evaluating the Biochemical and Haematological Safety of the Histoplasma capsulatum var. farciminosum ‘8ZH’ Vaccine in Foals
Source: Vet Med Sci. 2026 Jan 7;12(1):e70764. doi: 10.1002/vms3.70764 (PMC12779009; doi:10.1002/vms3.70764)
Supplement: Supplementary file 1 — Supplementary Figure 1. Western blot analysis of inactivated Histoplasma capsulatum var. farciminosum (‘8ZH’) antigen, Lane 1: Protein molecular weight marker (kDA); Lanes 2,3: Batch 1 (technical replicates); Lanes 4,5: Batch 2 (technical replicates); Lanes 6,7: Batch 3 (technical replicates) Supplementary Table 1. Statistical analysis of biochemical parameters Supplementary Table 2. Statistical analysis of hematological parameters (p‐values, 95% CI, and effect sizes (η2) for all hematological parameters.) [file VMS3-12-e70764-s001.docx]

Supplementary data


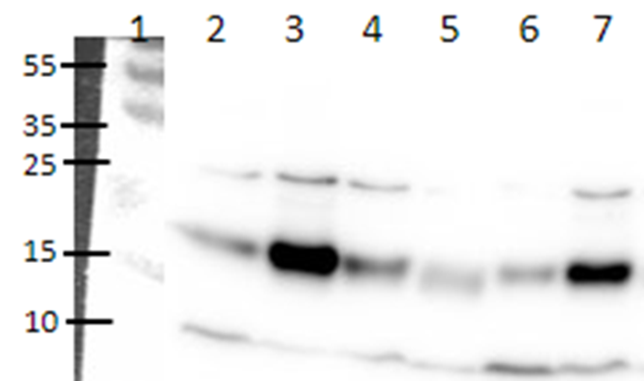


Supplementary figure 1. Western blot analysis of inactivated *Histoplasma capsulatum* var. *farciminosum* (‘8ZH’) antigen, Lane 1: Protein molecular weight marker (kDA); Lanes 2,3: Batch 1 (technical replicates); Lanes 4,5: Batch 2 (technical replicates); Lanes 6,7: Batch 3 (technical replicates)

Supplementary Table 1**.** Statistical analysis of biochemical parameters 
*(P-values, 95% CI, and effect sizes (η²) for all biochemical parameters.)*

| Parameter | P-Value | 95% CI | Effect Size (η²) |
| --- | --- | --- | --- |
| Total Protein | 0.03 | [67.5, 70.9] | 0.24 |
| Albumin | 0.06 | [30.8, 32.9] | 0.13 |
| Globulins | 0.07 | [27.0, 29.4] | 0.12 |
| Fibrinogen | 0.08 | [1.7, 2.6] | 0.11 |
| Serum Amyloid A (SAA) | 0.09 | [8.1, 10.2] | 0.10 |
| Haptoglobin | 0.07 | [31.5, 36.2] | 0.13 |
| Total Bilirubin | 0.04 | [23.8, 27.4] | 0.17 |
| AST | 0.04 | [208.5, 222.3] | 0.21 |
| ALT | 0.05 | [4.2, 5.6] | 0.16 |
| GGT | 0.17 | [22.5, 25.5] | 0.09 |
| CRP | 0.002 | [12.1, 22.7] | 0.31 |
| Urea | 0.05 | [3.1, 4.5] | 0.15 |
| Glucose | 0.08 | [3.6, 4.5] | 0.14 |
| Creatinine | 0.07 | [136.2, 140.7] | 0.18 |
| Cholesterol | 0.08 | [2.1, 2.9] | 0.15 |

Supplementary Table 2**.** Statistical analysis of hematological parameters 
*(P-values, 95% CI, and effect sizes (η²) for all hematological parameters.)*

| Parameter | P-Value | 95% CI | Effect Size (η²) |
| --- | --- | --- | --- |
| WBC | 0.02 | [8.8, 10.4] | 0.27 |
| RBC | 0.06 | [9.1, 9.6] | 0.16 |
| Hemoglobin | 0.07 | [135.5, 141.5] | 0.14 |
| Hematocrit | 0.07 | [41.2, 43.5] | 0.16 |
| Neutrophils | 0.03 | [55.4, 58.3] | 0.22 |
| Lymphocytes | 0.08 | [38.2, 41.5] | 0.13 |
| Monocytes | 0.07 | [4.1, 4.9] | 0.14 |
